# Sequence analysis and characterization of pyruvate kinase from *Clonorchis sinensis*, a 53.1-kDa homopentamer, implicated immune protective efficacy against clonorchiasis

**DOI:** 10.1186/s13071-017-2494-9

**Published:** 2017-11-09

**Authors:** Tingjin Chen, Hongye Jiang, Hengchang Sun, Zhizhi Xie, Pengli Ren, Lu Zhao, Huimin Dong, Mengchen Shi, Zhiyue Lv, Zhongdao Wu, Xuerong Li, Xinbing Yu, Yan Huang, Jin Xu

**Affiliations:** 1 Department of Parasitology, Zhongshan School of Medicine, Sun Yat-sen University, 74 Zhongshan 2nd Road, Guangzhou, Guangdong 510080 China; 2 Key Laboratory for Tropical Diseases Control, Sun Yat-sen University, Ministry of Education, Guangzhou, Guangdong 510080 China; 3 Provincial Engineering Technology Research Centre for Diseases-vectors Control, Guangzhou, Guangdong 510080 China; 4 Department of Clinical Laboratory, Third Affiliated Hospital, Sun Yat-sen University, Guangzhou, Guangdong 510630 China; 5 Department of Clinical Laboratory, Second Affiliated Hospital of Guangzhou Medical University, Guangzhou, Guangdong 510260 China

**Keywords:** *Clonorchis sinensis*, Pyruvate kinase, Pentamer, Expression profile, Excretory/secretory products, Immune response, Drug target, Vaccine candidate

## Abstract

**Background:**

*Clonorchis sinensis*, the causative agent of clonorchiasis, is classified as one of the most neglected tropical diseases and affects more than 15 million people globally. This hepatobiliary disease is highly associated with cholangiocarcinoma. As key molecules in the infectivity and subsistence of trematodes, glycolytic enzymes have been targets for drug and vaccine development. *Clonorchis sinensis* pyruvate kinase (*Cs*PK), a crucial glycolytic enzyme, was characterized in this research.

**Results:**

Differences were observed in the sequences and spatial structures of *Cs*PK and PKs from humans, rats, mice and rabbits. *Cs*PK possessed a characteristic active site signature (IKLIAKIENHEGV) and some unique sites but lacked the N-terminal domain. The predicted subunit molecular mass (Mr) of *Cs*PK was 53.1 kDa. Recombinant *Cs*PK (r*Cs*PK) was a homopentamer with a Mr. of approximately 290 kDa by both native PAGE and gel filtration chromatography. Significant differences in the protein and mRNA levels of *Cs*PK were observed among four life stages of *C. sinensis* (egg, adult worm, excysted metacercaria and metacercaria), suggesting that these developmental stages may be associated with diverse energy demands. *Cs*PK was widely distributed in adult worms. Moreover, an intense Th1-biased immune response was persistently elicited in rats immunized with r*Cs*PK. Also, rat anti-r*Cs*PK sera suppressed *C. sinensis* adult subsistence both in vivo and in vitro.

**Conclusions:**

The sequences and spatial structures, molecular mass, and expression profile of *Cs*PK have been characterized. r*Cs*PK was indicated to be a homopentamer. Rat anti-r*Cs*PK sera suppressed *C. sinensis* adult subsistence both in vivo and in vitro. *Cs*PK is worthy of further study as a promising target for drug and vaccine development.

**Electronic supplementary material:**

The online version of this article (10.1186/s13071-017-2494-9) contains supplementary material, which is available to authorized users.

## Background

*Clonorchis sinensis*, the causative agent of clonorchiasis, predominantly occurs in East Asia, including China, parts of Russia, South Korea, and northern Vietnam. Globally, it has been estimated that over 200 million people are at risk for clonorchiasis and that more than 15 million persons, of whom 13 million are in China, are infected [1]. Clonorchiasis represents a major tropical disease that is currently neglected [2]. *Clonorchis sinensis* is considered a group I carcinogen and an important risk factor for cholangiocarcinoma, a fatal malignant tumour of the bile duct that is usually associated with poor prognosis. It has been estimated that as many as 5000 cholangiocarcinoma cases resulting from clonorchiasis may occur yearly for decades to come [2–5]. During long-term infections, the excretory/secretory products (ESPs) of the worms are continuously discharged into the host. Therefore, as molecules that participate in host-parasite interactions, ESPs have been targeted for vaccine and drug development [6–8].

Transcriptomic and genomic data show that the enzymes that participate in the glycolytic pathway and the Krebs cycle are expressed during *C. sinensis* infection [9, 10]. Exogenous glucose is transported in the musculature of *C. sinensis* adults and is broken down during glycolysis, yielding lactic acid as a major end product and providing metabolic intermediates as well as energy [11, 12]. Glycolytic enzymes are extremely pertinent to the infectivity and subsistence of trematodes, and they have been well characterized as potential targets for drug and vaccine development [6, 13–15].

As a central metabolic regulator in most species, pyruvate kinase (PK, EC: 2.7.1.40, ATP: pyruvate phosphotransferase) catalyses the final rate­limiting reaction in glycolysis, essentially irreversibly transforming phosphoenolpyruvate (PEP) to pyruvate with the formation of one molecule of ATP. Additionally, both the substrates and the products of PK are involved in many biosynthetic pathways as well as in energy metabolism, placing it at a crucial metabolic intersection. In many parasites, including *Leishmania mexicana*, *Trypanosoma brucei*, *Toxoplasma gondii*, *Plasmodium falciparum* and *Schistosoma mansoni*, PK is highly regulated, indicating that it may play crucial regulatory roles in glycolysis in these parasites [15–19]. PK is considered a compelling therapeutic target for malignant tumours as well as for human pathogens such as apicomplexans [20]. Nearly all PKs exist as homotetramers [18, 21].

At present, research on the crucial glycolytic enzymes of *C. sinensis* is even more limited than research on the glycolytic enzymes of protozoa. Although PKs from *L. mexicana* [15, 22, 23], *T*. *gondii* [17, 20, 24], *T. brucei* [16], *Trypanosoma cruzi* [22], *P. falciparum* [18], *Eimeria tenella* [25], and *Entamoeba histolytica* [26] have been characterized in detail, the characteristics of *C. sinensis* PK (*Cs*PK) as a potential target for drug and vaccine development are unknown.

*Cs*PK is a 53.1-kDa homopentamer that possesses a characteristic active site signature (IKLIAKIENHEGV) as well as some unique sites but lacks the characteristic PK N-terminal domain. The mRNA levels, protein levels and tissue distribution of *Cs*PK at various life stages of *C. sinensis* were characterized. A T helper cell (Th) 1-biased immune response was induced in r*Cs*PK-immunized rats. Rat anti-r*Cs*PK sera suppressed *C. sinensis* adult subsistence in vivo and in vitro. This research indicates that *Cs*PK may be a promising candidate for the development of drugs and vaccines for clonorchiasis.

## Methods

### Sequence analysis

By using nucleotide sequence of PK gene (identify the query sequence), since they had used BLASTx algorithm (http://www.ncbi.nlm.nih.gov/), amino acid sequences of PK were retrieved from GenBank including that of *C. sinensis* (GAA54498.1). The physicochemical parameters and conserved domains of *Cs*PK were analysed using bioinformatics tools (ProtParam, InterProScan) in ExPASy (http://www.expasy.org/). The linear B cell epitopes were analysed with BepiPred (http://tools.immuneepitope.org/main/). The putative tertiary structure of *Cs*PK was simulated by comparative modelling using SWISS-MODEL (https://swissmodel.expasy.org/); the resulting structure was viewed using Swiss-Pdb Viewer and further evaluated using the Q-MEAN server. A phylogenetic tree of the PKs was constructed using the neighbour-joining method in MEGA version 5. Tests of phylogeny were conducted using the Bootstrap method with 1000 replications. Protein weight matrix was performed using Gonnet. The amino acid substitution model was constructed using the p-distance method.

### Expression and purification of recombinant *Cs*PK (r*Cs*PK)

The open reading frame (ORF) of *Cs*PK was amplified by the polymerase chain reaction (PCR) from the total cDNA of adult *C. sinensis* using specific primers (F: 5′-GCA CGG ATC CAT GCT ACA GAA GAT GA-3′, R: 5′-ATA GTC GAC AAA GCG GCT GAA GCT C-3′). The PCR products were purified and cloned into the prokaryotic expression vector pET-28a(+) (Novagen, Darmstadt, Germany). The recombinant plasmids were confirmed by digestion using the corresponding restriction enzymes and identified by DNA sequencing.

The recombinant plasmids were transformed into *Escherichia coli* BL21 (DE3) (Promega, Madison, USA). After being induced with 1 mM isopropyl-β-D-thiogalactopyranoside (Sigma-Aldrich, St. Louis, USA) at 37 °C for 6 h, the transformed cells were collected by centrifugation and sonicated on ice. r*Cs*PK was overexpressed as inclusion bodies. The sediment containing the His-tagged recombinant protein (r*Cs*PK) was washed with washing buffer (50 mM Tris-HCl, 50 mM NaCl, 1% Triton X-100, 1 mM EDTA, 2 M urea, pH 8.0) and dissolved in dissolving buffer (50 mM Tris-HCl, 50 mM NaCl, 1% Triton X-100, 1 mM EDTA, 6 M urea, pH 8.0) followed by centrifugation. The inclusion bodies dissolved readily in 6 M urea. The supernatant was applied to a Ni^2+^-NTA affinity column and eluted with a gradient of 5–300 mM imidazole containing 6 M urea. The protein eluted at 80–300 mM imidazole with high purity. The purified protein was renatured by sequential dialysis against solutions containing decreasing concentrations of urea (6 M to 0 M). Finally, the renatured protein was dialysed against phosphate-buffered saline (PBS).

The purified protein was subjected to electrophoresis on a 12% sodium dodecyl sulphate-polyacrylamide gel (SDS-PAGE) and identified by mass spectrometry (MS). Peptide mass spectra were performed on an ABI 4800 Proteomics Analyzer MALDI-TOF/TOF (Applied Biosystems, Md., USA). Both the MS and tandem mass spectrometry (MS/MS) data were interpreted and processed using the GPS Explorer software (version 3.6; Applied Biosystems, Md., USA) to match the protein name.

### Preparation of parasites, total worm extracts, *Cs*ESPs and antisera against r*Cs*PK/*Cs*ESPs

*Clonorchis sinensis* metacercariae were collected from experimentally infected freshwater *Ctenopharyngodon idellus* fish at our laboratory’s pool [27]. Viability and integrity of metacercariae were assessed under a microscope. 0.001% trypsin (Promega) in physiological saline was employed as an excystation stimulus in vitro. After activation, some encysted metacercariae was immediately collected in RIPA lysis buffer containing 1 mM phenylmethanesulfonyl fluoride (PMSF; Bioteke, Beijing, China) for extraction of total protein. Moreover, some encysted metacercariae were immediately collected for extraction of total RNA using TRIzol (Invitrogen, California, USA). Each SD rat was intragastrically infected with 50 metacercariae. At week 8 post-infection, the rats were sacrificed, and adult *C. sinensis* were recovered from their bile ducts.

Total worm extracts, *Cs*ESPs and mouse anti-*Cs*ESPs sera were obtained as described previously [6]. Briefly, living adult *C. sinensis* were cultured in DMEM (Gibco) under 5% CO_2_ at 37 °C. *Cs*ESPs were collected from the culture medium, treated with 1 mM PMSF, centrifuged and dialyzed in PBS. Living adult *C. sinensis* were crushed to prepare total worm extracts, stored at -80 °C. Rats and mice were subcutaneously immunized with 200/50 μg purified r*Cs*PK emulsified with an equivalent volume of complete Freund’s adjuvant (Sigma-Aldrich) followed by 2 boosters of r*Cs*PK (100/25 μg) emulsified with an equivalent volume of incomplete Freund’s adjuvant (Sigma-Aldrich) at 2-week intervals. Preimmune sera were harvested before the first immunization. Immune sera were harvested at 2-week intervals 2 to 24 weeks after immunization.

### Determination of the apparent molecular mass (Mr) of r*Cs*PK

Purified r*Cs*PK was subjected to native PAGE on 8% gels. The Mr. of r*Cs*PK was confirmed by determining the relationship between the log Mr. (kDa) of the standard marker proteins (Sigma-Aldrich) and their elution volumes (Ve) on AKTA FPLC using Sepharose 12 10/300 GL gel filtration chromatography (GFC) (GE Healthcare, Pittsburgh, USA) [28]. The GFC column was run at a flow rate of 0.8 ml/min in 50 mM Tris-HCl containing 100 mM KCl (pH 7.5). Based on the deduced equation, the Mr. of r*Cs*PK was derived using the obtained Ve of r*Cs*PK.

### Western blotting

Purified r*Cs*PK (2.5 μg), *Cs*ESPs (25 μg) and total worm extract (25 μg) were separated by 12% SDS-PAGE and electrotransferred to a PVDF membrane in a Trans-Blot transfer cell (Bio-Rad, Hercules, USA) at 100 V for 1 h. The PVDF membranes were blocked with 5% skim milk in PBS (pH 7.4) at 4 °C overnight and then incubated with a mouse His-tagged monoclonal antibody (1:2000 dilution, Novagen, Darmstadt, Germany), mouse anti-r*Cs*PK serum (1:2000 dilution), serum of mice infected with *C. sinensis* (1:2000 dilution), mouse anti-*Cs*ESP serum (1:2000 dilution) or preimmune mouse serum (1:2000 dilution) at RT for 2 h. After 3 washes in PBS, the membranes were incubated with HRP-conjugated goat anti-mouse IgG (1:2000 dilution) at RT for 1 h. After washing the membranes 5 times, detection was performed using chemiluminescence.

### *Cs*PK mRNA and protein levels at various life stages of *C. sinensis*

Total RNAs were isolated from the egg, adult, excysted metacercaria and metacercaria of *C. sinensis* using TRIzol (Invitrogen). *Clonorchis sinensis* β-actin (accession No. EU109284) was used as an internal control, which was amplified with specific primers 5′-ACC GTG AGA AGA TGA CGC AGA-3′ and 5′-GCC AAG TCC AAA CGA AGA ATT-3′. Real-time PCR was performed using the SYBR Premix Ex *Taq* Kit (Takara, Shiga, Japan) in the iQ5 Real-Time PCR Detection System (Bio-Rad, Hercules, USA) using specific primers 5′-AAG ATA AGG CAG ATT TAC GCT-3′ and 5′-CTG GGA TTT CAA TAC CAAGAT-3′. The PCR program was 95 °C for 5 min followed by 40 cycles of 95 °C for 10 s and 56 °C for 30 s. Melting curves were obtained by heating the samples to 95 °C for 30 s and 60 °C for 15 s followed by an increase in temperature to 95 °C. Semiquantitative analysis was conducted according to the 2^-ΔΔCT^ method [29, 30].

Western blotting was used to study the levels of *Cs*PK protein present at the above 4 life stages of *C. sinensis*. Parasites from those stages were suspended in RIPA lysis buffer containing 1 mM PMSF. A supernatant was obtained by centrifugation at 10,000 × *g* for 20 min at 4 °C. Total protein (40 μg) of each stage was subjected to 12% SDS-PAGE and blotted onto PVDF membrane. The blots were incubated with sera from mice immunized with r*Cs*PK (1:200 dilution) or with mouse preimmune sera (1:200 dilution) followed by incubation with HRP-conjugated goat anti-mouse IgG (1:2000 dilution). Detection was performed with chemiluminescence. Relative protein levels were quantitated using Tanon Gis software (Tanon 4100, Shanghai, China).

### Immunolocalization of *Cs*PK in *C. sinensis*

Adults and metacercariae of *C. sinensis* were fixed in formalin, embedded in paraffin wax and cut into 5-μm-thick sections. After successive deparaffinization with xylene and hydration in a graded ethanol series, the sections were blocked with normal goat serum at RT for 2 h. The sections were then incubated with mouse anti-r*Cs*PK serum (1:100 dilution) in a humidified chamber overnight at 4 °C. Mouse preimmune serum diluted at the same ratio was used as a negative control. The sections were washed 3 times with PBS containing 0.05% Tween-20 (PBS-T, pH 7.4) and 2 times in PBS followed by incubation with Cy3-conjugated goat anti-mouse IgG (1:400 dilution, Molecular Probes, USA) in the dark at RT for 1 h. BSA (0.1%) in PBS was used as the dilution buffer. The sections were imaged using a fluorescence microscope (Leica, Wetzlar, Germany).

### Enzyme-linked immunosorbent assay (ELISA) of antibody titres and isotypes of IgG elicited by r*Cs*PK

Microplates were incubated overnight at 4 °C with 2.5 μg/well-purified r*Cs*PK in coating buffer (0.1 M carbonate-bicarbonate, pH 9.6). The plates were then blocked with 5% skim milk in PBS-T at 37 °C for 2 h. The protein bound to the wells was incubated with serial dilutions of the immune sera (obtained at week 6 following the first immunization) elicited by r*Cs*PK followed by washing. Serum from rats or mice immunized with PBS was used as a negative control. HRP-conjugated goat anti-host specific IgG (1:20,000 dilution in 0.1% BSA PBS-T, Proteintech, Rosemont, USA) was used as the secondary antibody. After incubation for 1 h and 3 washes in PBS-T, 100 μl of substrate solution (TMB, BD Biosciences, San Diego, USA) was added to each well followed by incubation for 12 min in the dark. After the addition of 2 M H_2_SO_4_ to terminate the reaction, the absorbance at 450 nm was measured. The levels of total IgG and IgG isotypes in sera obtained at various time points (2, 4, 6, 8, 10, 12, 14, 16, 18, 20, 22 and 24 weeks after the first immunization) were also measured. The primary antibodies were diluted 1:400. HRP-conjugated goat anti-rat IgG (1:20,000 dilution) and IgG1/IgG2a (1:10,000 dilution, Bethyl, TX, USA) were used as secondary antibodies.

### Immune protection conferred by r*Cs*PK

Thirty-two 6-week-old SD rats were randomly separated into 4 groups: an adjuvant group, an infection group, a r*Cs*PK group, and a PBS group (*n* = 8 for each group). The SD rats in the r*Cs*PK and PBS groups received subcutaneous injections of either 200 μg r*Cs*PK or an equal volume of PBS, respectively, emulsified with complete Freund’s adjuvant. Two boosters of 100 μg r*Cs*PK or an equal volume of PBS emulsified with incomplete Freund’s adjuvant were given at 2-week intervals. The SD rats in the adjuvant group received subcutaneous injections of an equal volume of adjuvant. The SD rats in the infection group received no pretreatment.

After measurement of the animals’ antibody titres at 6 weeks after immunization, all rats were challenged by oral administration of 80 live *C. sinensis* metacercariae. The eggs per gram faeces (EPG) were calculated at week 6 post-infection. For worm burden evaluation, the rats were sacrificed 8 weeks post-infection, and adult *C. sinensis* were recovered from their livers. The EPG and worm burden were calculated blindly. The reduction in the parasite burden was calculated as follows: egg reduction rate (%) = [(average EPG of control group – average EPG of experimental group) / average EPG of control group] × 100%; worm reduction rate (%) = [(average worm burden of control group – average worm burden of experimental group) / average worm burden of control group] × 100% [31].

### Culture of *C. sinensis* adults in the presence of rat anti-r*Cs*PK sera

*Clonorchis sinensis* adults were recovered from infected rats, washed 4 times with sterilized PBS containing 1% antibiotics (streptomycin 100 U/ml and penicillin 100 μg/ml) and transferred to 12-well plates at 20 adults per well in 2 ml/well of low-glucose DMEM containing 1% antibiotics. Serum from rats that had been immunized with r*Cs*PK or preimmune serum from the same animals was mixed with the medium at dilutions of 1:40–1:160. Low-glucose DMEM was used as the blank control. At 1–10, 15, 18, 20, 22, 24, 26 and 28 days of incubation, the adult worms were counted and monitored for 5 min using a microscope (Leica). Adults that failed to display pumping and muscle contraction after 5 continuous mechanical stimuli were classified as dead [32].

### Statistics and software

All experiments were performed at least 3 times. SPSS software (version 17.0; SPSS, Inc., IL, USA) was employed for statistical analysis. Differences in IgG isotypes and immune protection between the groups were analysed using Student’s *t*-test. The survival rates of cultured adults were analysed using the Kaplan-Meier method, and differences between the groups were determined using the log-rank (Mantel-Cox) test. The results of the analyses are expressed as the mean ± standard deviation; significance was set at a *P*-value < 0.05.

## Results

### Sequence analysis and putative spatial structure of *Cs*PK

**Fig. 1:**
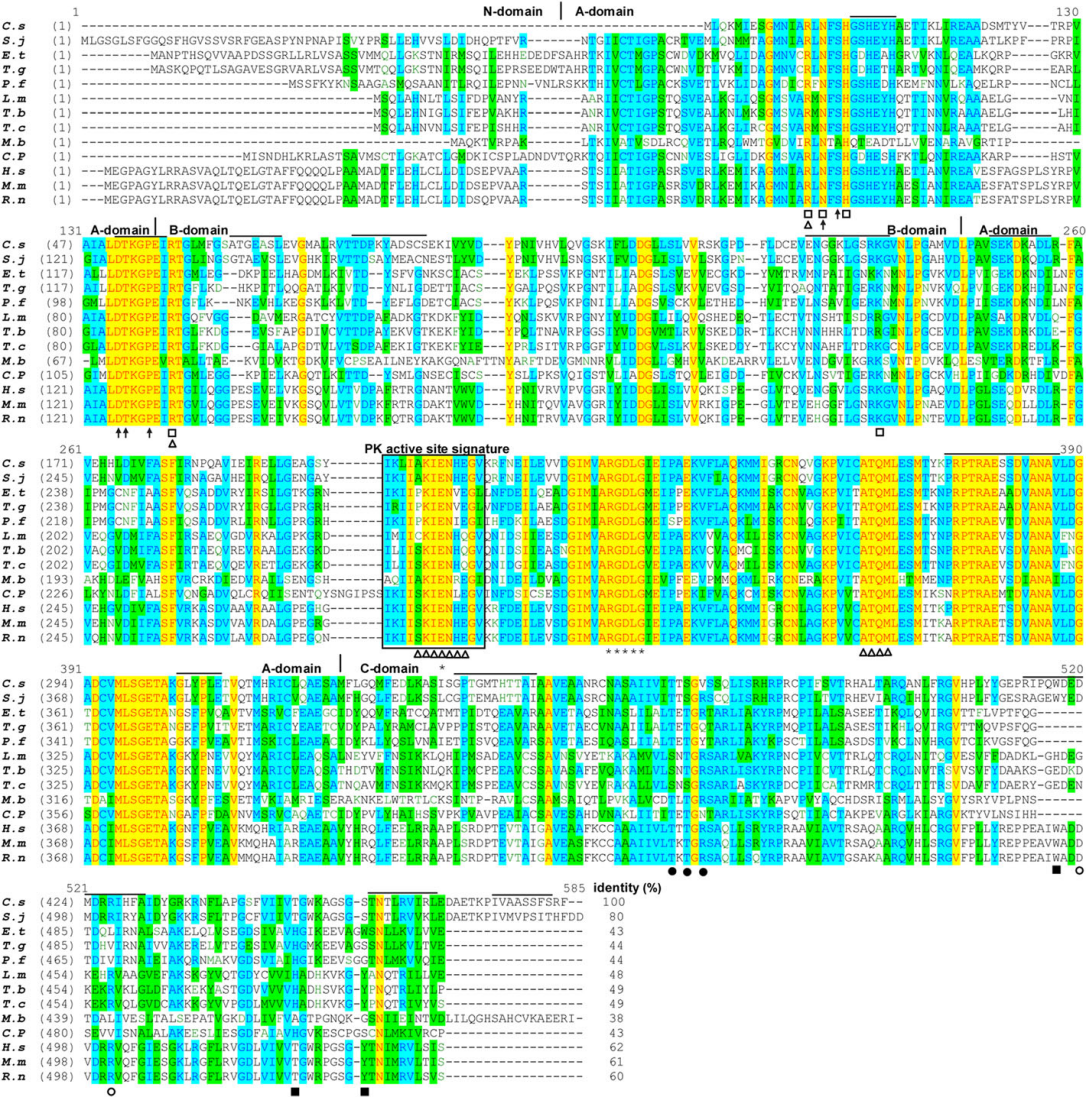
Sequence analysis of *Cs*PK. An alignment of the protein sequence of *Cs*PK with those of PKs from other organisms is shown. *Clonorchis sinensis* (*C.s*, GAA54498.1), *Schistosoma japonicum* (*S.j*, AAW27129.1), *Eimeria tenella* (*E.t*, AAC02529.1), *Toxoplasma gondii* (*T.g*, BAB47171.1), *Plasmodium falciparum* (*P.f*, CAD50538.1), *Leishmania mexicana* (*L.m*, CAA52898.2), *Trypanosoma brucei* (*T.b*, P30615.1), *Trypanosoma cruzi* (*T.c*, EKG02834.1), *Mastigamoeba balamuthi* (*M.b*, AAK94944.1), *Cryptosporidium parvum* (*C.p*, 4DRS_A), *Homo sapiens* (*H.s*, AAA60104.1), *Mus musculus* (*M.m*, NP_001093249.1), and *Rattus norvegicus* (*R.n*, AAA41880.1). The 3D–domains (N/A/B/C) are marked with vertical lines. The 22 predicted B cell linear epitopes with more trustworthiness are indicated by the black lines above the alignment. The rectangle indicates the PK active site signature. The triangles and open squares indicate the PEP and ADP binding sites, respectively. The binding sites of the sugar, 1-phosphate and 6-phosphate moieties of the allosteric activator fructose-1,6-bisphosphate (F16BP) are indicated by closed squares, open circles and closed circles, respectively. The black arrows and asterisks indicate monovalent cation and divalent cation binding sites, respectively

The ORF of *Cs*PK is 1458 bp in length and encodes 485 amino acids (aa). In its protein sequence, *Cs*PK shows the identities to the PK of *Opisthorchis viverrini* (99%), followed *Schistosoma japonicum* (80%), *Brugia malayi* (59%), *Hymenolepis microstoma* (39%) and *Echinococcus granulosus* (33%). The protein sequence included the characteristic active site signature (IKLIAKIENHEGV) (Fig. 1). This sequence, which occurs between amino acid residues 203 and 215 of *Cs*PK, contains a lysine (K) residue that forms part of the catalytic site and that appears to be responsible for interconverting enolpyruvate and pyruvate. L205 was unique to *Cs*PK, replacing the usual isoleucine (I). A207 of *Cs*PK replaced the usual serine (S) found in the rat, mouse and human enzymes (Additional file 1: Figure S1). The consensus regions for the binding of PEP, ADP, and monovalent as well as divalent cations were well conserved.

In the sequence alignment, the 4 three-dimensional (3D) domains described for PK are shown. The N-terminal domain, which only appears in eukaryotic PKs, is very long in *S. japonicum*, *T*. *gondii* and *E. tenella* but absent in *C. sinensis*. Domains A and B, which contain the catalytic sites, are well conserved. In contrast, domain C, which contains the effector sites and is localized at the C-terminal end, is diverse [25]. *Cs*PK contains 22 predicted linear B cell epitopes and lacks a transmembrane region and a signal peptide. *Cs*PK was predicted to be expressed in the cytosol [24].

The 3D structure of *Cs*PK was simulated using SWISS-MODEL based on the molecular model of truncated *Tg*PK1 (an N-terminal truncated version, Protein Data Bank PDB: 3GG8 [20]), which exhibited 44.67% identity to *Cs*PK. The GMQE score of 0.74 and the Q-MEAN4 of -0.83 supported the quality of the model. *Cs*PK consisted only of domain A (blue), domain B (red) and domain C (green). The catalytic sites appear at the interface of domains A and B (Additional file 1: Figure S1). It is notable that the K454-S459 region and the G413-D423 loop, which corresponds to the mobile loop in yeast PK [33], are components of the allosteric binding site [20].

In human PK-M2 (PDB: 3BJF [34]) and *Cs*PK, the F16BP binding sites, which interact with the phosphate moieties, are also conserved with different conformations. The ribbon drawing shows a superposition between domain A of *Cs*PK with the closed rabbit PK-M1 in complex with ATP and the inhibitor oxalate (PDB: 1A49 [35]). The significant residues of the two proteins are highly conserved and show diverse conformations.

In the active site of *Cs*PK and in that of LmPYK-suramin (PDB: 3PP7 [22]) complexed with glycerol and suramin (an inhibitor of *T. brucei* glycolytic enzymes), the significant residues are highly conserved and exhibit the same conformation.

**Fig. 2:**
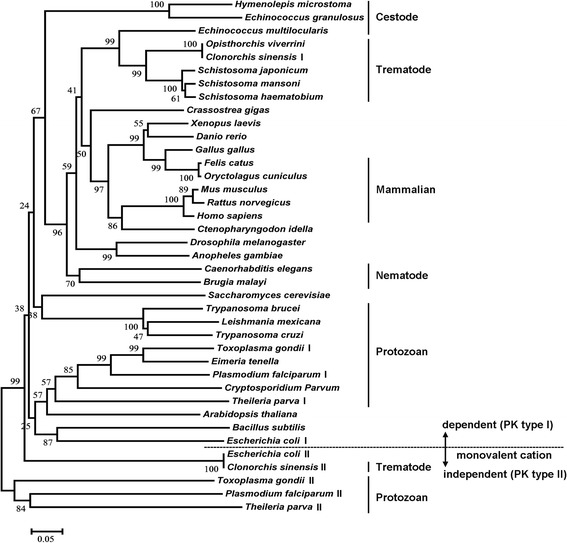
Neighbour-joining phylogenetic tree of PKs. The bootstrap values are displayed at the branching point (test of phylogeny by the bootstrap method with 1000 replications). The bar indicates the substitution by p-distance method. The protein sequences were obtained from GenBank and DDBJ. The sequences are as follows: *Escherichia coli* (AAA24392.1, AAA24473.1), *Bacillus subtilis* (P80885.2), *Arabidopsis thaliana* (BAB10461.1), *Cryptosporidium parvum* (4DRS_A), *Plasmodium falciparum* (CAD50538.1, AAN35560), *Eimeria tenella* (AAC02529.1), *Toxoplasma gondii* (BAB47171.1, KFH06835.1), *Trypanosoma brucei* (P30615.1), *Trypanosoma cruzi* (EKG02834.1), *Leishmania mexicana* (CAA52898.2), *Theileria parva* (XP_764242.1, XP_764703.1), *Saccharomyces cerevisiae* (CAA24631.1), *Brugia malayi* (XP_001898626.1), *Hymenolepis microstoma* (CDS33796.1), *Echinococcus granulosus* (CDS23463.1), *Echinococcus multilocularis* (CDS43052.1), *Caenorhabditis elegans* (CAA93424.2), *Anopheles gambiae* (EAA10555.6), *Drosophila melanogaster* (AAC16244.1), *Crassostrea gigas* (CAJ28914.1), *Clonorchis sinensis* (GAA54498.1, GAA58090.1), *Opisthorchis viverrini* (KER20867.1), *Schistosoma japonicum* (AAW27129.1), *Schistosoma haematobium* (KGB40466.1), *Schistosoma mansoni* (CCD76479.1), *Danio rerio* (NP_955365.1), *Xenopus laevis* (NP_001084341.1), *Gallus gallus* (NP_990800.1), *Felis catus* (P11979.2), *Homo sapiens* (AAA60104.1), *Mus musculus* (NP_001093249.1), *Rattus norvegicus* (AAA41880.1), *Ctenopharyngodon idella* (AFY98078.1)

In the phylogenetic tree of PKs (Fig. 2), *Cs*PK grouped very closely with the PKs of trematodes. *Cs*PK was closely related to PKs of vertebrates, followed by those of nematodes and cestodes, whereas it was distantly related to the PKs of protozoa. Phylogenetic, sequence and structural analysis revealed that *Cs*PK (T52 and E56) is related to the group of monovalent cation-dependent isozymes (type I) [24]. In contrast, *Cs*PKII (GAA58090.1, 245 aa) clusters with *E. coli* PKII (AAA24473.1), along with three isozymes from the protozoans *T. gondii* (KFH06835.1), *P. falciparum* (AAN35560) and *T. parva* (XP_764703.1). These results suggest a different evolutionary origin of the two isozymes in *C. sinensis*.

### Expression and apparent Mr. of r*Cs*PK

**Fig. 3:**
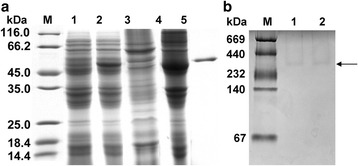
Expression and apparent Mr. of r*Cs*PK. Proteins were visualized by Coomassie Blue staining. Lane M contains protein molecular weight markers. **a** Expression and purification of r*Cs*PK. Lysate of *E. coli* transformed with pET-28a(+)-*Cs*PK without induction (Lane 1) and with induction (Lane 2); supernatant (Lane 3) and precipitate (Lane 4) of lysate of *E. coli* with pET-28a(+)-*Cs*PK with induction; and purified r*Cs*PK (Lane 5). **b** In Lane M, the protein bands with known Mr. (in descending order) are thyroglobulin (669 kDa), ferritin (440 kDa), catalase (232 kDa), lactate dehydrogenase (140 kDa), and BSA (67 kDa). Lane 1, Freshly purified r*Cs*PK; Lane 2, r*Cs*PK stored for 4 weeks at -80 °C with 4 cycles of freezing and thawing

The putative theoretical isoelectric point of *Cs*PK is 6.69, and its predicted subunit Mr. is 53.1 kDa. Purified r*Cs*PK containing a 6× His-tag displayed a single band of approximately 58.0 kDa, consistent with the calculated Mr. (Fig. 3a, Lane 5). The peptides obtained from the purified protein, which was demonstrated by MS analysis, were matched to those of *Cs*PK at a protein coverage of 20% (data not shown).

Native PAGE and molecular sieve chromatography were used to determine the protein’s multimeric status. In native PAGE (Fig. 3b), the mobility of r*Cs*PK was between that of ferritin (440 kDa) and that of catalase (232 kDa). According to the equation describing the electrophoretic profile, the Mr. of r*Cs*PK was approximately 290 kDa.In GFC, r*Cs*PK eluted as a single peak between β-amylase (200 kDa) and ferritin (440 kDa) (Additional file 2: Figure S2).

### Western blotting

**Fig. 4:**
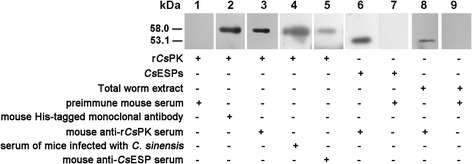
Western blotting of r*Cs*PK after SDS-PAGE. Blots containing r*Cs*PK were incubated with preimmune mouse serum (Lane 1), a mouse His-tagged monoclonal antibody (Lane 2), mouse anti-r*Cs*PK sera (Lane 3), sera from mice infected with *C. sinensis* (Lane 4), or mouse anti-*Cs*ESPs sera (Lane 5). Blots containing *Cs*ESPs were incubated with mouse anti-r*Cs*PK sera (Lane 6) or with preimmune mouse serum (Lane 7). Blots containing total worm extract were incubated with mouse anti-r*Cs*PK sera (Lane 8) or with preimmune mouse serum (Lane 9)

r*Cs*PK was recognized by a mouse His-tagged monoclonal antibody, mouse anti-r*Cs*PK sera, sera of mice infected with *C. sinensis* and mouse anti-*Cs*ESPs sera; in all cases, a cross-reactive band of approximately 58.0 kDa (including the Mr. of the His-tag) was noted. This immunoreactive band did not appear on blots that had been probed with naive mouse sera. Additionally, *Cs*ESPs and total worm extracts probed with mouse anti-r*Cs*PK sera, but not with preimmune sera, showed an immunoreactive band of approximately 53.1 kDa (Fig. 4).

### mRNA and protein levels and immunolocalization of *Cs*PK at the life stages of *C. sinensis*

**Fig. 5:**
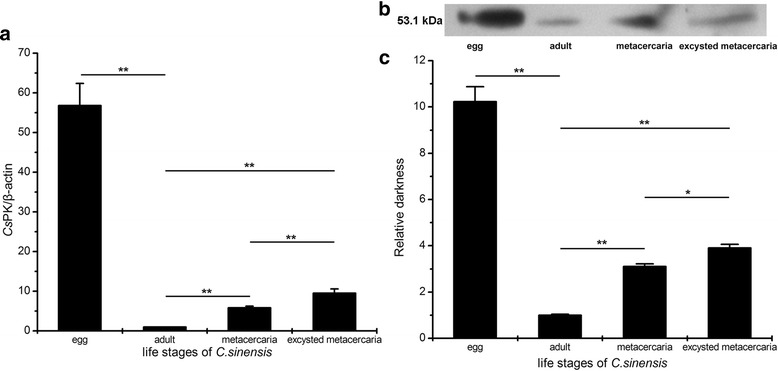
mRNA and protein levels of *Cs*PK at various life stages of *C. sinensis*. **a** Real- time PCR. The β-actin mRNA of *C. sinensis* was used as an internal control. Semiquantitative analysis was conducted using the 2^-ΔΔCt^ method. Significant differences in the mRNA levels of *Cs*PK in egg, adult, excysted metacercaria, and metacercaria were observed (*P* < 0.01). The mRNA level of *Cs*PK in egg was higher than that in adult (56.79-fold, *t*_(2)_ = 17.392, *P* = 0.003), metacercaria (9.72-fold, *t*_(2.02)_ = 15.844, *P* = 0.004) and excysted metacercaria (5.97-fold, *t*_(4)_ = 14.477, *P* < 0.001). **b** Western blotting. Total protein (40 μg) in extracts obtained at each life stage was probed with mouse anti-r*Cs*PK sera, revealing specific immunoreactive protein bands at approximately 53.1 kDa. No corresponding band was detected with preimmune mouse serum (data not shown). **c** Relative protein levels were analysed using Tanon Gis software. The protein level of *Cs*PK was maximal in eggs, followed by excysted metacercaria, metacercaria, and adults. The protein levels were consistent with the mRNA levels. (*P < 0.05; **P < 0.01; egg *vs* adult: *t*_(4)_ = 12.950, *P* < 0.001; excysted metacercaria *vs* adult: *t*_(4)_ = 16.542, *P* < 0.001; metacercaria *vs* adult: *t*_(4)_ = 13.951, *P* < 0.001; excysted metacercaria *vs* metacercaria: *t*_(4)_ = -3.680, *P* = 0.021)

The mRNA levels of *Cs*PK were measured at the adult, egg, excysted metacercaria, and metacercaria stages of *C. sinensis* (Fig. 5a). Significant differences were observed in the mRNA levels of *Cs*PK at the above 4 life stages (*P* < 0.01). The mRNA level of *Cs*PK in the egg was higher than that in the adult (56.79-fold, *t*_(2)_ = 17.392, *P* = 0.003), metacercaria (9.72-fold, *t*_(2.02)_ = 15.844, *P* = 0.004) and excysted metacercaria (5.97-fold, *t*_(4)_ = 14.477, *P* < 0.001).

A specific polypeptide of molecular weight approximately 53.1 kDa was detected with mouse anti-r*Cs*PK sera by Western blotting analysis. The protein level of *Cs*PK was highest in the egg, followed by excysted metacercaria, metacercaria and adult (Fig. 5b, c). No corresponding band was detected when preimmune mouse serum was used (data not shown).

**Fig. 6:**
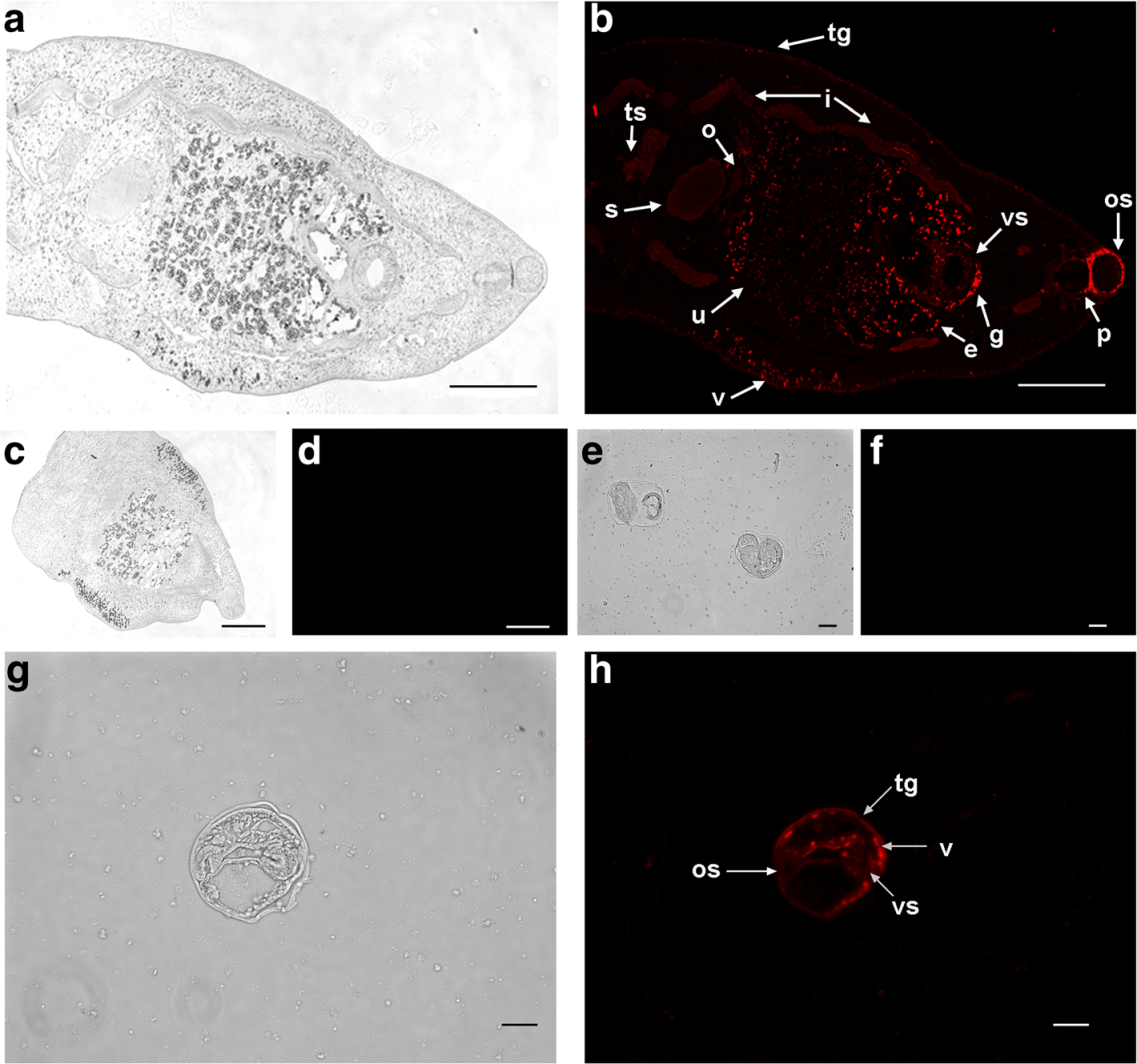
Immunolocalization of *Cs*PK in *C. sinensis*. Mouse anti-r*Cs*PK sera and Cy3-conjugated goat anti-mouse IgG were used as the primary and secondary antibodies, respectively. Preimmune mouse serum was used as the primary antibody for the negative controls. Panels (**c**), (**d**), (**e**), and (**f**) show negative controls. Panels (**b**), (**d**), (**f**), and **h** are fluorescence microscopic images; the same areas of the samples photographed under white light are shown in panels (**a**), (**c**), (**e**), and **g** with *scale-bars*. Panel (**b**), localization of *Cs*Pk in adults; panel **h**, localization of *Cs*Pk in metacercariae. *Abbreviations:* tg, tegument; e, egg; v, vitellarium; os, oral sucker; vs, ventral sucker; g, genital pore; s, seminal receptacle; i, intestine; ts, testicle; u, uterus;o, ovary; p, pharynx. *Scale-bars*: **a**-**d**, 100 μm; **e**-**h**, 10 μm

In sections of adult worms (Fig. 6), strong fluorescence due to *Cs*PK was observed in the oral sucker, ventral sucker, pharynx, genital pore, vitellarium, tegument, intestine, seminal receptacle, testicle, ovary, uterus and in eggs within the uterus. Moreover, strong fluorescence was apparent on the tegument, oral sucker, ventral sucker, and vitellarium, in metacercariae. No specific fluorescence was observed in negative controls incubated with preimmune mouse serum.

### IgG isotypes and immune protective efficacy elicited by r*Cs*PK

**Fig. 7:**
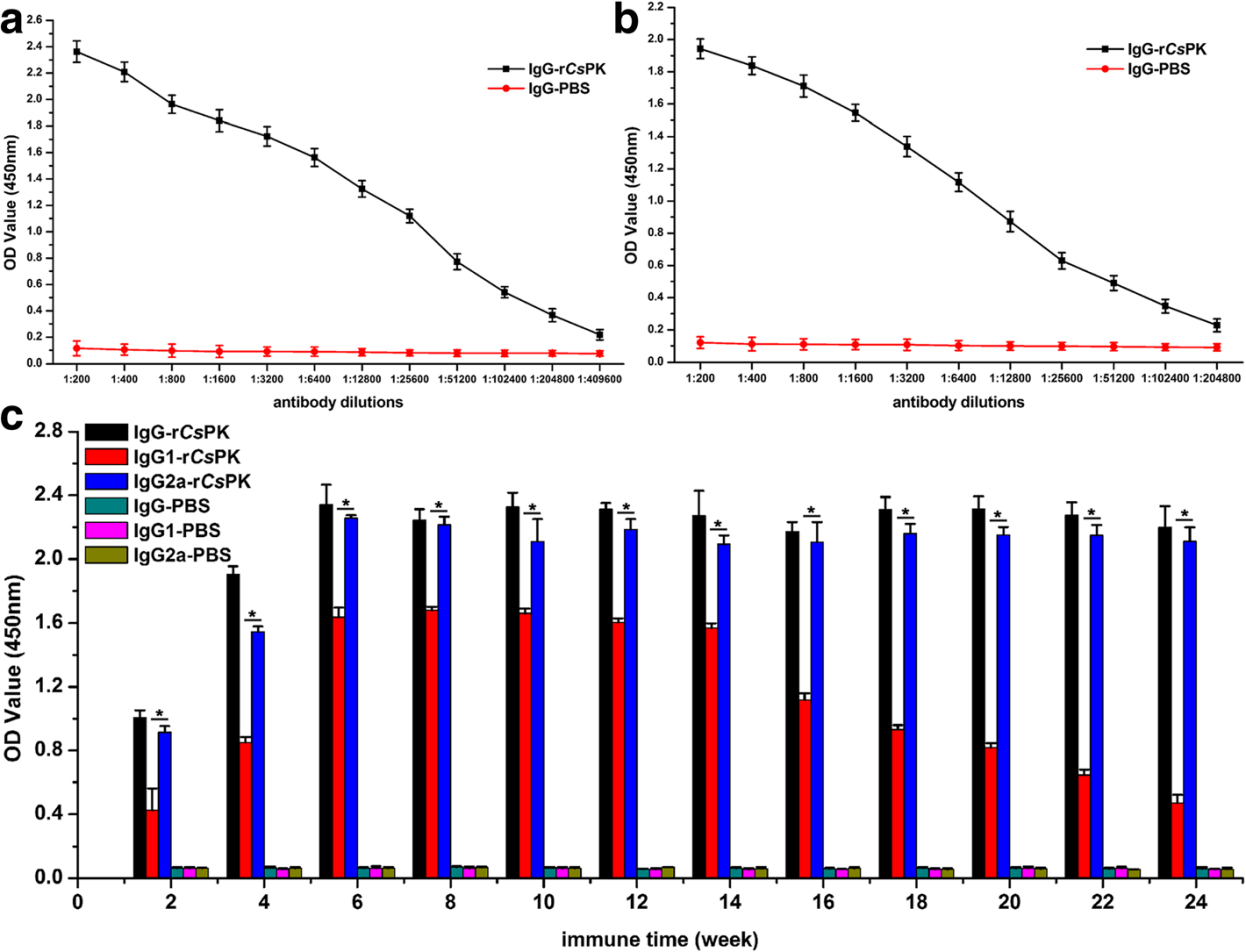
ELISA determination of antibody titres and isotypes of IgG elicited by r*Cs*PK. Antibody titres of IgG elicited by r*Cs*PK in rats (**a**) and mice (**b**). IgG isotypes elicited by r*Cs*PK in rats (**c**). *P ≦ 0.001. 2 week: *t*_(6)_ = 6.886, *P* < 0.001; 4 week: *t*_(6)_ = 27.959, *P* < 0.001; 6 week: *t*_(6)_ = 19.829, *P* < 0.001; 8 week: *t*_(6)_ = 19.278, *P* < 0.001; 10 week: *t*_(6)_ = 6.264, *P* = 0.001; 12 week: *t*_(6)_ = 17.319, *P* < 0.001; 14 week: *t*_(6)_ = 16.977, *P* < 0.001; 16 week: *t*_(6)_ = 15.057, *P* < 0.001; 18 week: *t*_(6)_ = 37.271, *P* < 0.001; 20 week: *t*_(6)_ = 48.557, *P* < 0.001; 22 week: *t*_(6)_ = 40.796, *P* < 0.001; 24 week: *t*_(6)_ = 32.550, *P* < 0.001

The titre of anti-r*Cs*PK IgG in rats and mice was as high as 1:409,600 to 1:204,800 at week 6 post-immunization, indicating that the protein is strongly immunogenic (Fig. 7a, b). In rat anti-r*Cs*PK sera, IgG2a and IgG1 levels increased at week 2 and reached peak values at 6 and 8 weeks. IgG1 levels decreased markedly after 16 weeks, whereas IgG2a levels were maintained at a high level until 24 weeks. Between 2 and 24 weeks post-immunization, the level of IgG2a was significantly higher than that of IgG1 (Fig. 7c).

The EPG values of the PBS group, the adjuvant group, the infection group, and the r*Cs*PK group were 3983.3 ± 386.7; 4075.0 ± 473.0; 3895.8 ± 424.1; and 1716.7 ± 230.3, respectively. The numbers of worms recovered from the above 4 groups were 25.1 ± 4.8; 24.8 ± 5.3; 26.1 ± 5.1; and 11.2 ± 2.5, respectively (Table 1). The EPG and worm burden showed a marked decrease in the r*Cs*PK group compared with the control groups (*t*_(11.410)_ = 14.245, *P* < 0.001; *t*_(14)_ = 7.299, *P* < 0.001). The egg reduction rate and worm reduction rates were 56.90% and 55.22% (compared with the PBS group), respectively. No significant difference in EPG or worm burden was observed among the PBS, adjuvant, and infection groups.

**Table 1:** EPG and worm burden of rats in diverse groups

Group (*n*=8)	EPG^a^	Worm burden^a^
PBS	3983.3 ± 386.7	25.1 ± 4.8
adjuvant	4075.0 ± 473.0 (*t*_(14)_ = -0.424, *P* = 0.678)	24.8 ± 5.3 (*t*_(14)_ = 0.149, *P* = 0.884)
infection	3895.8 ± 424.1 (*t*_(14)_ = 0.431, *P* = 0.673)	26.1 ± 5.1 (*t*_(14)_ = -0.407, *P* = 0.690)
r*Cs*PK	1716.7 ± 230.3 (*t*_(11.410)_ = 14.245, *P* < 0.001)	11.2 ± 2.5 (*t*_(14)_ = 7.299, *P* < 0.001)

^a^Compared with the PBS group

### Rat anti-r*Cs*PK sera affects *C. sinensis* adult subsistence in vitro

**Fig. 8:**
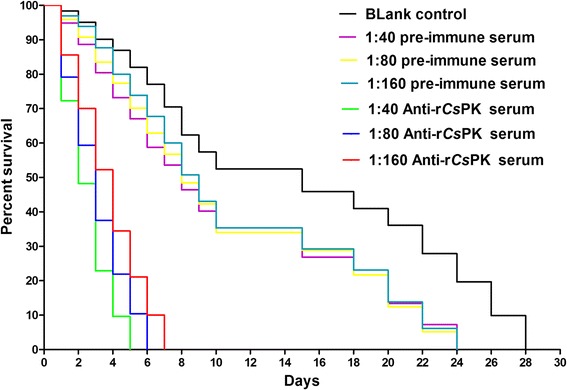
Rat anti-r*Cs*PK sera inhibits *C. sinensis* adult subsistence in vitro. The median subsistence times of *C. sinensis* adults in the blank control group, the 1:40 preimmune serum group, the 1:80 preimmune serum group, the 1:160 preimmune serum group, the 1:40 anti-r*Cs*PK serum group, the 1:80 anti-r*Cs*PK serum group, and the 1:160 anti-r*Cs*PK serum group were 15, 8, 8, 9, 2, 3 and 4 days, respectively. No significant difference in the rate of survival of the preimmune serum groups was observed at any serum dilution (1:40 preimmune serum group *vs* 1:80 preimmune serum group: *χ* ^2^ = 0.01289, *df* = 1, *P* = 0.9096; 1:80 preimmune serum group *vs* 1:160 preimmune serum group: *χ* ^2^ = 0.09872, *df* = 1, *P* = 0.7534; 1:40 preimmune serum group *vs* 1:160 preimmune serum group: *χ* ^2^ = 0.1657, *df* = 1, *P* = 0.6839). There were significant differences among the other groups in the rate of survival (1:40 anti-r*Cs*PK serum group *vs* 1:80 anti-r*Cs*PK serum group: *χ* ^2^ = 8.058, *df* = 1, *P* = 0.0045; 1:80 anti-r*Cs*PK serum group *vs* 1:160 anti-r*Cs*PK serum group: *χ* ^2^ = 8.092, *df* = 1, *P* = 0.0044; blank control group *vs* 1:40 preimmune serum group: *χ* ^2^ = 16.15, *df* = 1, *P* < 0.0001; blank control group *vs* 1:80 preimmune serum group: *χ* ^2^ = 15.54, *df* = 1, *P* < 0.0001; blank control group *vs* 1:160 preimmune serum group: *χ* ^2^ = 11.39, *df* = 1, *P* = 0.0007)

The median survival times of *C. sinensis* adults in the blank control group, in preimmune sera groups (with 1:40, 1:80 or 1:160 dilution), and in anti-r*Cs*PK sera groups (with 1:40, 1:80 or 1:160 dilution) were 15, 8, 8, 9, 2, 3 and 4 days, respectively (Fig. 8). No significant difference in survival rate was observed among preimmune sera groups with different dilution (*P* > 0.05). There were dominant differences of survival rates among all other groups (*P* < 0.01).

## Discussion

In the present research, we investigated and compared the sequence and spatial structure of *Cs*PK with those of PKs from humans, rats, mice, and rabbits, all of which are definitive hosts of *C. sinensis. Cs*PK is a 53.1-kDa homopentamer PK that lacks the characteristic N-terminal domain but possesses a characteristic active site signature (IKLIAKIENHEGV) and some unique sites. The mRNA levels, protein levels and immunolocalization of *Cs*PK at various life stages of *C. sinensis* were characterized. *Cs*PK was a component of *Cs*ESPs. Also, a Th1-biased immune response was elicited in rats immunized with r*Cs*PK. Furthermore, rat anti-r*Cs*PK sera suppressed *C. sinensis* adult subsistence both in vivo and in vitro.

The protein sequence and spatial structure analysis revealed that *Cs*PK possesses the typical characteristics of a PK (Fig. 1, Additional file 1: Figure S1). To our knowledge, a homopentamer PK from any organism has not previously been reported. The peptides of r*Cs*PK were analysed by MS analysis (data not shown). Based on SDS-PAGE, the Mr. of r*Cs*PK was approximately 58 kDa (including a His-tag) (Fig. 3b). A band of approximately 58 kDa in r*Cs*PK reacted with a mouse His-tagged monoclonal antibody (Fig. 4), whereas the Mr. of r*Cs*PK was approximately 290 kDa, as confirmed by native PAGE and GFC (Fig. 3, Additional file 2: Figure S2). The above results indicate that *Cs*PK is a homopentamer, whereas almost all PKs from other species are homotetrameric enzymes [18, 21].

The X-ray crystallographic structures of some PKs of diverse organisms (such as *E. coli*, *L. mexicana*, yeast, rabbit muscle, human, and cat) are highly similar with respect to ordinary PK topology [23, 34–39]. PKs are homotetrameric enzymes consisting of identical subunits of approximately 50–60 kDa; each subunit consists of 3 to 4 domains: N (a variable N-terminal domain), A, B, and C [23, 40]. The N-terminal helical domain is only present in eukaryotic PKs and can be removed from human erythrocyte PK without affecting the enzyme’s activity or stability [39]. The N-terminal domain is very long in *S. japonicum* but was absent in *C. sinensis*.

In each subunit, the catalytic sites lie at the interface of domains A and B, and the allosteric sites are located in domain C (Fig. 1, Additional file 1: Figure S1) [20, 35, 37]. Multiple alignment of the protein sequences of PKs from parasitic species with those of human, rat, and mouse PKs (Fig. 1) showed that the known binding sites of PEP, cations and ADP, which lie within domains A and B, are well conserved (47–100%), whereas the effector binding sites, which lie within domain C, are more divergent in some species. In this research, domain A was found to be much more highly conserved (27%) than domains B (10%) and C (3%). This result is consistent with results obtained for PKs from various species [41]. Interestingly, in the characteristic active site signature of *Cs*PK (IKLIAKIENHEGV), L205 and A207 replace the usual isoleucine (I) and serine (S) found in the rat, mouse and human enzymes. Phylogenetic analysis revealed marked differences between *Cs*PK and PKs from the definitive hosts of *C. sinensis*, cat, rat, human and mouse (Fig. 2).

In contrast to the similarities in the sequence motifs found in the A and B domains, the putative sites for effector binding located in domain C, which participate in the allosteric regulation of PKs, are not conserved between parasite and host isoforms. In addition, some sites that are important for effector binding in domain C and L205 in the characteristic active site signature of the PKs are unique to *Cs*PK (Fig. 1). The findings reported above are particularly interesting with respect to the development of selective and specific inhibitors of parasite PK. Despite the presence of identical catalytic sites and the high sequence conservation (95%) of the M1-spliced isoform, selective targeting of human PK-M2 that included only the region involved in allosteric regulation was shown to offer a chance of targeting cellular metabolism for the treatment of malignant tumours [40, 42]. This provides proof of principle that PKs might be selectively targeted despite the presence of high degrees of homology among the enzymes from various species [40].

In human PK-M2 and *Cs*PK, the F16BP binding sites, which interact with phosphate moieties [34], are very conservative with different conformations (Additional file 1: Figure S1). In the active sites of *Cs*PK and LmPYK-suramin complexed with glycerol and suramin (an inhibitor of *T. brucei* glycolytic enzymes) [22], the significant residues are highly conserved and display the same conformation (Additional file 1: Figure S1). These observations offer new insight into the mechanism of regulation and the functional features of *Cs*PK as well as into its potential as a drug target for clonorchiasis.

r*Cs*PK reacted with mouse anti-r*Cs*PK sera by Western blotting, revealing its good immunoreactivity. The recognition of r*Cs*PK by sera of mice infected with *C. sinensis* hints that *Cs*PK might be a component of circulating antigens [43, 44]. Additionally, r*Cs*PK reacted with mouse anti-*Cs*ESPs sera. Furthermore, *Cs*ESPs were recognized by mouse anti-r*Cs*PK sera, showing a band of approximately 53.1 kDa. These results demonstrate that *Cs*PK is also a component of ESPs.

As the key glycolytic enzyme, *Cs*PK was widely distributed in the tissues of adult worm, suggesting that *Cs*PK is significant for *C. sinensis*. The locations in which *Cs*PK was found included the pharynx, the intestine, and the tegument, tissues from which ESPs are released [6]. In trematodes, the tegument functions as a dynamic organ that participates in mutual interactions between the parasite and host. Trematode tegument is composed of the syncytial membrane that is highly active in nutrient uptake. The presence of *Cs*PK in ESPs might result from the shedding and recondition of the tegument [45]. The trematode intestine is a site of nutrient absorption and digestion as well as an important source of ESPs [46]. With the addition of its distribution in tegument, as occurs in fodinichnia, *Cs*PK may be involved in obtaining a sustainable energy supply through the digestion and absorption of glucose from the host.

The localization of *Cs*PK within the musculature, including the pharynx, the oral sucker, and the ventral sucker, may be related to energy demands for adhesion behaviour as well as muscular contraction. Its localization in generative organs, including the ovary, vitellarium, uterus, testis, genital pore, and seminal receptacle, hints that the enzymatic activity of *Cs*PK in glucose metabolism in the above organs may be needed to satisfy the energy requirements of *C. sinensis* for growth and reproduction. The vitellarium of the trematode makes a critical contribution to egg production by providing nutrients to the oosperm, providing eggshell material, and related catalytic activities [47].

Bioinformatics analysis indicated that *Cs*PK contains numerous predicted T cell as well as B cell epitopes. The high levels of specific antibodies induced by r*Cs*PK may be attributed to its variety of B cell epitopes. It is well known that IgG1 and IgG2a are elicited by Th2 and Th1, respectively. These data suggest that r*Cs*PK elicits Th1-biased immunoreaction.

During long-term infection, Th1 to Th2 shift has been shown to occur and to result in long-term persistence of adult worms as well as chronic clonorchiasis [48]. Th1 cells direct protective pro-inflammatory immune responses [49]. Experiments have shown that the levels of IgG2 and IgG1 antibodies in protected animals are 100-fold and 10-fold higher than those of unprotected animals. The protective effect is strongly associated with the avidity, and the titre of IgG2 antibodies elicited [50, 51]. In addition, an effective Th1 immunoreaction is essential for successful vaccine inoculation against a majority of viral as well as bacterial pathogens [52]. The Th1-biased immune response observed for *Cs*PK revealed its high immunogenicity and might account for its protective efficacy.

Nucleic acid-based, as well as protein-based vaccines for clonorchiasis, have been reported. Some DNA vaccines elicit Th1-biased immune responses, whereas other DNA vaccines and protein vaccines elicit combined Th1/Th2 immune responses [1]. In contrast, r*Cs*PK elicited a Th1-biased immune response. A majority of proteins that are regarded as vaccine candidates are ESPs or tegumental proteins. *Cs*PK was identified as a component of ESPs and was shown to be deposited on the tegument of adult worms and metacercariae. Rat anti-r*Cs*PK sera inhibited *C. sinensis* adult subsistence in vivo and in vitro. Our results indicate that *Cs*PK may be a promising candidate for the development of vaccines for clonorchiasis.

## Conclusions

*Cs*PK is a 53.1-kDa homopentamer PK without a characteristic N-terminal domain; it possesses a characteristic active site signature (IKLIAKIENHEGV) and some unique sites. *Cs*PK was widely distributed in the tissues and organs of adult worms, including the oral sucker, ventral sucker, pharynx, genital pore, vitellarium, tegument, intestine, seminal receptacle, testicle, ovary, uterus and in eggs within the uterus. It was shown to be a component of *Cs*ESPs. r*Cs*PK possesses strong immunogenicity as well as immunoreactivity. r*Cs*PK provoked a Th1-biased immune response. In addition, rat anti-r*Cs*PK sera suppressed *C. sinensis* adult subsistence in vivo and in vitro. This research reveals that *Cs*PK might be a promising vaccine candidate and that it has potential as a drug target against *C. sinensis* infection. The selective development of specific inhibitors against *Cs*PK and its immune protective mechanisms are worthy of further study.

## Additional files

Additional file 1: Figure S1. Putative tertiary modelling of CsPK. K+ and Mg2+ ions are shown as grey and black spheres, respectively. The N-terminal domain is shown in black. **a** Ribbon drawing of superposed structure models of *Cs*HK (darker tone) and truncated *Tg*PK1 (lighter tone). The A, B, and C domains of *Cs*HK are shown in blue, red and green, respectively. The catalytic site at the interface of domains A and B and the allosteric site in domain C are highlighted. **b** Ribbon representation of F16BP (red stick) binding sites of human PK-M2 (lighter tone). S434, S437 (yellow stick), W482 (magenta stick), and R489 (orange stick), which interact with the phosphate moieties, are indicated. The putative corresponding structure of *Cs*PK (darker tone) is shown in panel **c**. In the active site signature of PK, I267 and S269 (dark red sticks) are replaced by L205 and A207 (blue-violet sticks) in *Cs*HK. Oxalate is indicated as a ball model. **d** Ribbon representation of the K^+^-PK-Mg^II^oxalate-Mg^II^ATP complex closed active site (rabbit PK-M1). ATP, oxalate, and significant residues are shown as red, magenta, and yellow sticks, respectively. **e** Ribbon drawing of the superposition between the A domains of *Cs*PK with the closed rabbit PK-M1 in complex with ATP and oxalate (dark and lighter tones, respectively). The corresponding significant residues of *Cs*PK are shown in orange (stick). K^+^ and Mg^2+^ ions, ATP and oxalate, are shown for reference, with their positions derived from a superposition with 1A49. **f** Ribbon drawing of superposed structural models of *Cs*PK (darker tone) and LmPYK-suramin (lighter tone) complexed with glycerol (magenta stick) and suramin (an inhibitor of *T. brucei* glycolytic enzymes, red stick). **g** Enlargement of the active site of the LmPYK-suramin structure. Significant residues are coloured yellow (stick). The putative corresponding structure of *Cs*PK is shown in panel **h**. The corresponding significant residues of *Cs*PK are coloured orange (stick). (TIFF 2964 kb)

Additional file 2: Figure S2. Apparent Mr. of r*Cs*PK. **a** Determination of the Mr. of r*Cs*PK using 8% native PAGE according to Fig. 3b. After plotting the HRm values of the standard markers against their Mrs., we drew a curve, fitted an equation to the curve, and used the equation for calculating the Mr. of r*Cs*PK. **b** Elution profile of r*Cs*PK in GFC. **c** Mr. of r*Cs*PK as detected with GFC. The calibration curve relating the elution volumes (Ve) and the log Mr. (kDa) of standard marker proteins were obtained with AKTA FPLC using a Sepharose 12 10/300 GL GFC column. Based on the deduced equation, the Mr. of r*Cs*PK was calculated from the obtained Ve of r*Cs*PK. (TIFF 252 kb)

## Abbreviations

3D: Three-dimensional
BCA: Bicinchoninic acid
BSA: Bovine sera albumin
*Cs*ESPs: ESPs of *C. sinensis*
*Cs*PK: *Clonorchis sinensis* pyruvate kinase
ELISA: Enzyme-linked immunosorbent assay
EPG: Eggs per gram faeces
ESPs: Excretory/secretory products
HRP: Horseradish peroxidase
Mr.: Molecular mass
MS: Mass spectrometry
ORF: Open reading frame
PBS: Phosphate-buffered saline
PCR: Polymerase chain reaction
PEP: Phosphoenolpyruvate
PK: Pyruvate kinase
SD: Sprague-dawley
Th: T helper cells
