# Supplementary figures and images for: Sequence analysis and characterization of pyruvate kinase from Clonorchis sinensis, a 53.1-kDa homopentamer, implicated immune protective efficacy against clonorchiasis
Source: Parasit Vectors. 2017 Nov 9;10:557. doi: 10.1186/s13071-017-2494-9 (PMC5680780; doi:10.1186/s13071-017-2494-9)

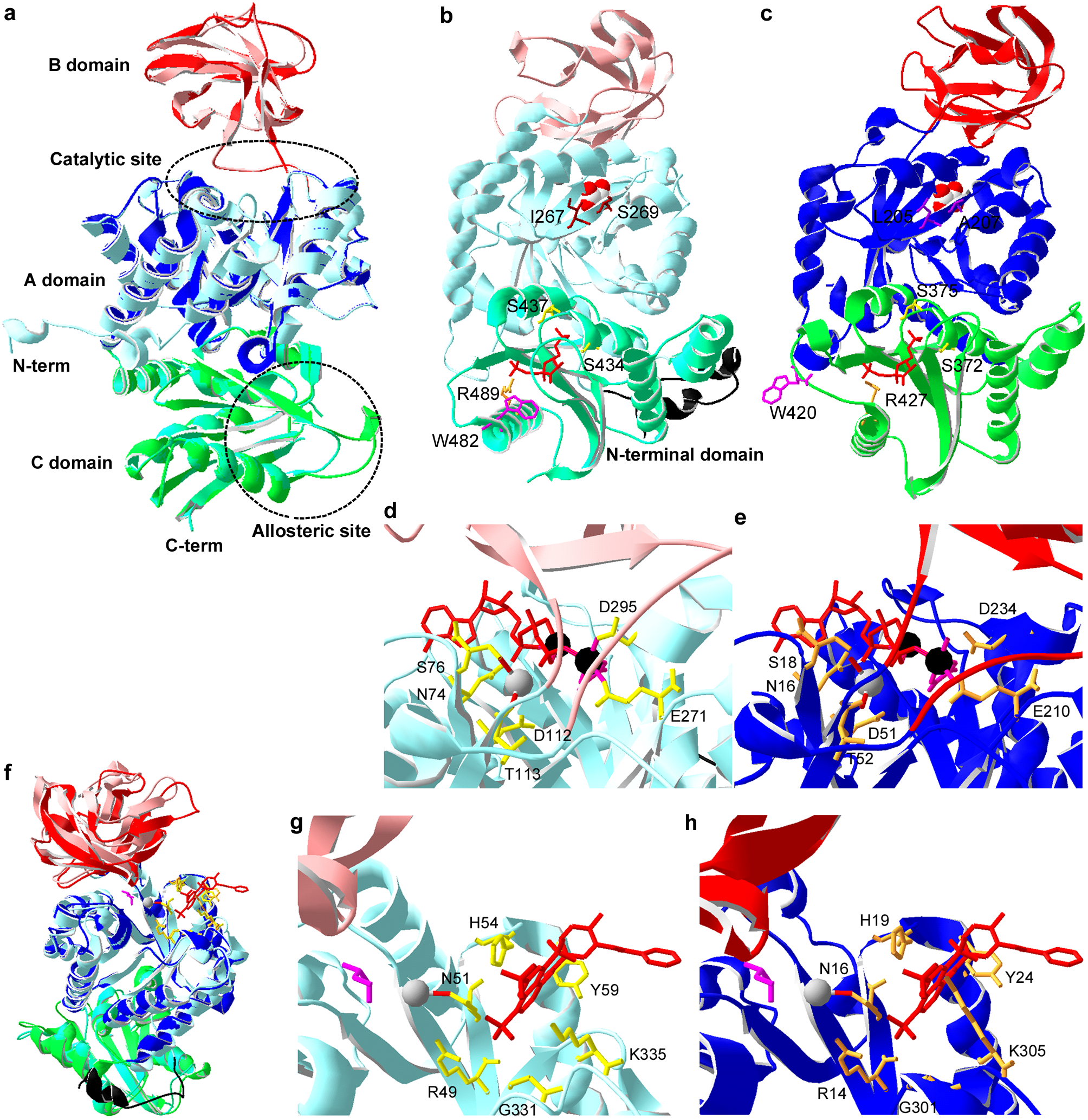

Supplement: Supplementary file 1 — Putative tertiary modelling of CsPK. K+ and Mg2+ ions are shown as grey and black spheres, respectively. The N-terminal domain is shown in black. a Ribbon drawing of superposed structure models of CsHK (darker tone) and truncated TgPK1 (lighter tone). The A, B, and C domains of CsHK are shown in blue, red and green, respectively. The catalytic site at the interface of domains A and B and the allosteric site in domain C are highlighted. b Ribbon representation of F16BP (red stick) binding sites of human PK-M2 (lighter tone). S434, S437 (yellow stick), W482 (magenta stick), and R489 (orange stick), which interact with the phosphate moieties, are indicated. The putative corresponding structure of CsPK (darker tone) is shown in panel c. In the active site signature of PK, I267 and S269 (dark red sticks) are replaced by L205 and A207 (blue-violet sticks) in CsHK. Oxalate is indicated as a ball model. d Ribbon representation of the K+-PK-MgIIoxalate-MgIIATP complex closed active site (rabbit PK-M1). ATP, oxalate, and significant residues are shown as red, magenta, and yellow sticks, respectively. e Ribbon drawing of the superposition between the A domains of CsPK with the closed rabbit PK-M1 in complex with ATP and oxalate (dark and lighter tones, respectively). The corresponding significant residues of CsPK are shown in orange (stick). K+ and Mg2+ ions, ATP and oxalate, are shown for reference, with their positions derived from a superposition with 1A49. f Ribbon drawing of superposed structural models of CsPK (darker tone) and LmPYK-suramin (lighter tone) complexed with glycerol (magenta stick) and suramin (an inhibitor of T. brucei glycolytic enzymes, red stick). g Enlargement of the active site of the LmPYK-suramin structure. Significant residues are coloured yellow (stick). The putative corresponding structure of CsPK is shown in panel h. The corresponding significant residues of CsPK are coloured orange (stick). (TIFF 2964 kb) [file 13071_2017_2494_MOESM1_ESM.tif]

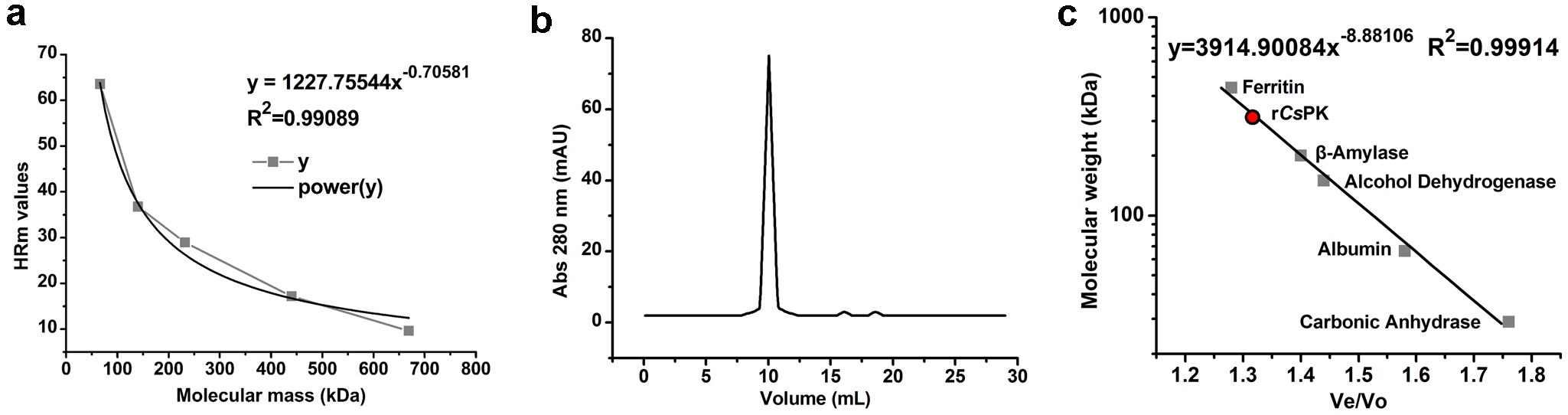

Supplement: Supplementary file 2 — Apparent Mr. of rCsPK. a Determination of the Mr. of rCsPK using 8% native PAGE according to Fig. 3b. After plotting the HRm values of the standard markers against their Mrs., we drew a curve, fitted an equation to the curve, and used the equation for calculating the Mr. of rCsPK. b Elution profile of rCsPK in GFC. c Mr. of rCsPK as detected with GFC. The calibration curve relating the elution volumes (Ve) and the log Mr. (kDa) of standard marker proteins were obtained with AKTA FPLC using a Sepharose 12 10/300 GL GFC column. Based on the deduced equation, the Mr. of rCsPK was calculated from the obtained Ve of rCsPK. (TIFF 252 kb) [file 13071_2017_2494_MOESM2_ESM.tif]
